# Effect of age on the gap-prepulse inhibition of the cortical N1-P2 complex in humans as a step towards an objective measure of tinnitus

**DOI:** 10.1371/journal.pone.0241136

**Published:** 2020-11-05

**Authors:** Yunseo Ku, Do Youn Kim, Chiheon Kwon, Tae Soo Noh, Moo Kyun Park, Jun Ho Lee, Seung Ha Oh, Hee Chan Kim, Myung-Whan Suh

**Affiliations:** 1 Department of Biomedical Engineering, College of Medicine, Chungnam National University, Daejeon, Korea; 2 Interdisciplinary Program in Bioengineering, Graduate School, Seoul National University, Seoul, Korea; 3 Department of Otorhinolaryngology- Head and Neck Surgery, Seoul National University Hospital, Seoul, Korea; 4 Interdisciplinary Program in Bioengineering, Graduate School, Department of Biomedical Engineering, College of Medicine, Seoul, Korea; 5 Institute of Medical & Biological Engineering, Medical Research Center, Seoul National University, Seoul, Korea; University of Michigan, UNITED STATES

## Abstract

The gap-prepulse inhibition of the acoustic startle reflex has been widely used as a behavioral method for tinnitus screening in animal studies. The cortical-evoked potential gap-induced inhibition has also been investigated in animals as well as in human subjects. The present study aimed to investigate the effect of age on the cortical N1-P2 complex in the gap-prepulse inhibition paradigm. Fifty-seven subjects, aged 20 to 68 years, without continuous tinnitus, were tested with two effective gap conditions (embedded gap of 50- or 20-ms duration). Retest sessions were performed within one month. A significant gap-induced inhibition of the N1-P2 complex was found in both gap durations. Age differently affected the inhibition, depending on gap duration. With a 50-ms gap, the inhibition decreased significantly with the increase in age. This age-inhibition relationship was not found when using a 20-ms gap. The results were reproducible in the retest session. Our findings suggest that the interaction between age and gap duration should be considered when applying the gap-induced inhibition of the cortical-evoked potential as an objective measure of tinnitus in human subjects. Further studies with tinnitus patients are warranted to identify gap duration that would minimize the effects of age and maximize the difference in the inhibition between those with and without tinnitus.

## Introduction

Tinnitus, which affects 10–20% of the world’s population, involves the perception of phantom sounds in the absence of a physical stimulus [1, 2]. In some cases, tinnitus might cause severe social and economic difficulties due to interference of the incessant sound with daily activities, causing degradation of the patients’ quality of life [3]. Despite its prevalence and debilitating effects, at present, there is no objective way to diagnose tinnitus. The current use of a patient’s subjective response makes the diagnosis unreliable and hinders the development of effective treatment [4].

The gap-prepulse inhibition of the acoustic startle (GPIAS) has been used as an objective way of screening tinnitus in animals [5–10]. GPIAS is the recording of the startle response, representing the brainstem-midbrain circuitry (subcortical pathway) [5, 11]. It is presented as a ratio between a response elicited by a sound pulse preceded by a silent gap embedded in continuous narrowband noise (gap response) and a response evoked by a sound pulse without the gap (no-gap response). Inhibition is elicited in the gap response because the silent gap acts as an inhibiting cue for the subsequent sound pulse. The use of the GPIAS paradigm for tinnitus assessment was originally based on the hypothesis that continuous tinnitus sound fills the silent gap and results in reduced inhibition of the gap response. Recent human studies have investigated the GPIAS paradigm, using eye blink or post-auricular muscle response as indicators of a startle response [12–14]. However, some animal studies reported results that contradict the “filling-in” hypothesis. For example, different GPIAS deficits were recorded, depending on the location of the silent gap [15, 16]. Similarly, in a study in humans, the GPIAS deficit in tinnitus patients occurred irrespective of the similarity between the frequency of the continuous noise and the tinnitus pitch [17]. The validity of GPIAS is still controversial and some researchers have casted doubt on the startle response used in the paradigm. Wilson et al. have pointed out that the whole-body startle reflex and eye-blinks are complex responses that respond not only to auditory stimulus but also to visual or somatosensory stimuli [14]. Furthermore, Berger et al. proposed that the startle response, which is primarily regulated by the brainstem circuit, may not be directly related to the auditory cortex [18]. Thus, finding a more reliable neural response related to tinnitus would be helpful [18, 19].

Although the pathogenesis of tinnitus is not fully understood, animal studies have shown that tinnitus is associated with increased synchrony in the dorsal cochlear nucleus, inferior colliculus, and the auditory cortex [7, 20, 21]. Furthermore, patients with tinnitus were observed to have changes in connectivity between the auditory and extra-auditory regions [22]. These findings are in line with the significant gap-prepulse inhibition (GPI) of the cortical auditory-evoked potential observed in animals and human subjects with tinnitus [18, 23]. The startle response and the N1-P2 complex of cortical-evoked potential showed different sensitivities of the prepulse inhibition (PPI) to drugs such as bromocriptine, and ketanserin or to acute tryptophan depletion [24–26]. These findings suggest that the PPI mechanism of cortical-evoked potentials is considered different from that of the startle response, but several studies have suggested that there might be some shared mechanisms between them [27, 28]. The major N1 and P2 components are believed to be originated from multiple anatomic sources in the primary auditory cortex and auditory association regions [29]. Since the cortical N1-P2 complex of the auditory late response (ALR) is known as an obligatory response to sensory inputs [30, 31], we considered it to be a good neural response that could reflect the GPI. A high test-retest reliability of the N1-P2 complex has been found in terms of measurement and sensory gating [23, 32–35]. In a previous study, we used the GPI paradigm to investigate the use of the N1-P2 complex for tinnitus assessment [36]. The peak-to-peak amplitudes ratios of the gap and no-gap responses were analyzed and compared between patients with tinnitus and age-matched healthy controls. The tinnitus group showed a GPI deficit with a 20-ms gap but not with 100- or 50-ms gaps. The “filling-in” hypothesis was not proved because the deficit occurred irrespective of the match between the tinnitus-pitch and the background frequency. These findings were consistent with a previous GPIAS study in humans [17]. Impaired cortical auditory processing has been proposed as a possible explanation for the GPI deficit associated with tinnitus.

Auditory temporal processing refers to the capacity to precisely detect the temporal features of sounds [37]. Various aspects, from sensory neuronal circuitry to cortical processing of sound information, are involved in the auditory processing ability [38, 39]. It has been known for a while that several factors other than tinnitus also affect the auditory temporal processing. One example is the effect of gap duration on the GPIAS, as a significant increase in inhibition has been reported with increase in gap duration up to 50 ms [12]. Moreover, as noted in several previous cognitive studies, aging can cause a decline in sensory abilities and auditory processing [40–43]. However, the effect of age on the GPI outcome with evoked potentials has not been investigated. Given this paucity of relevant work, the present study aimed to elucidate the influence of age on the N1-P2 complex when assessing 20 to 68-year-old subjects, using the GPI paradigm.

## Materials and methods

### Subjects

Sixty-seven adults (31 females) were recruited from August, 2015 to December, 2017 via poster ads or word of mouth in Seoul National University Hospital, Seoul, Korea. Our sample aimed to represent normal subjects of different ages and genders matched to the demographic characteristics of tinnitus patients. Structured histories were obtained to assess pre-existing medical conditions, with a focus on hearing and tinnitus. None of the subjects were found to have suffered from tinnitus or any other otological/neurological disorders. The inclusion criteria of hearing thresholds at 1 kHz and 8 kHz frequencies were ≤30 and ≤70 dB HL (hearing level), respectively. These thresholds were selected based on the output stimuli sound level and the maximum output limitation of the experimental equipment. To evaluate subjects’ eligibility, all subjects were tested by pure tone audiometry with standardized devices and methods (American National Standards Institute s3.6–2004) across six different frequencies (0.25, 0.5, 1, 2, 4, and 8 kHz). A commercial audiometer (AD629, Interacoustics, Denmark) was used for the pure tone audiometry. Subjects having hearing thresholds higher than the inclusion criteria (*n* = 6) and subjects who showed noisy ALR with unclear N1-P2 (*n* = 4) were excluded by eye inspection. A total of 57 subjects (29 females) with a mean age of 43.7 years (range, 20 to 68 years) participated in the test session. Of the 57 subjects, 46 (21 females) participated in a retest session; the mean time interval between test and retest sessions was 27 days (SD: 32.6). Details of the subject characteristics are presented in Table 1.

**Table 1:** Subject characteristics.

	Test	Retest
***n* (Females)**	57 (29)	46 (21)
**Age, years (SD)**	43.7 (14.6)	43.8 (15.2)
**Hearing threshold (dB HL)**		
**1 kHz (SD)**	8.3 (6.6)	9.4 (6.6)
**8 kHz (SD)**	17.8 (16.6)	18.4 (16.6)

SD, standard deviation; HL, hearing level.

The Institutional Review Board of the Seoul National University Hospital approved the present study (IRB No. H-1312-077-541), and all participants have signed a written informed consent form. This study followed the principles expressed in the 1964 Declaration of Helsinki and its later amendments.

### Sound stimuli

**Fig 1:**
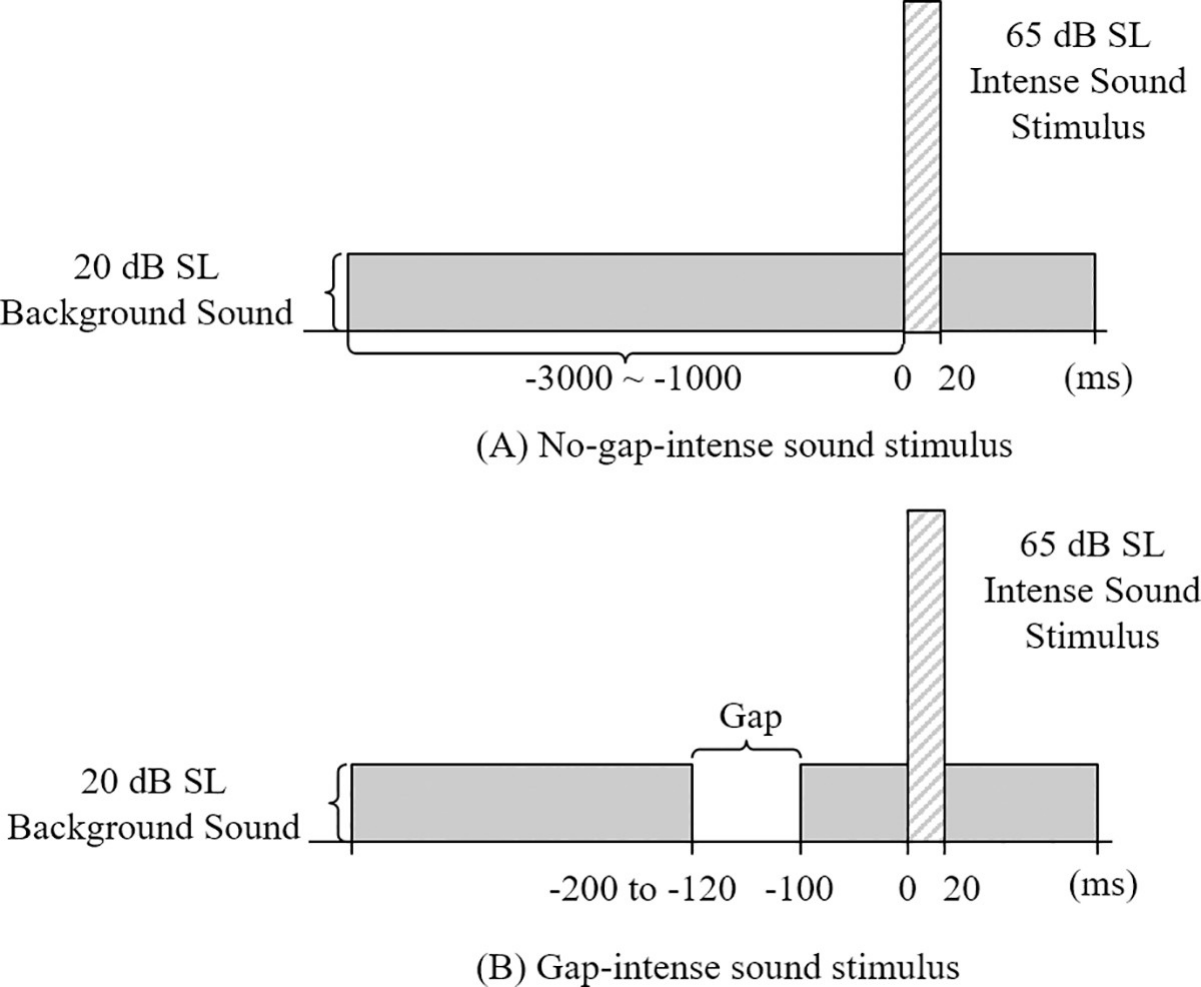
Diagrams of sound stimuli. (A) No-gap stimulus containing a background sound at an intensity of 20 dB SL (8 kHz pure tone) and a 20 ms long intense sound stimulus (1 kHz tone burst). (B) Gap stimulus containing a background sound at an intensity of 20 dB SL (8 kHz pure tone) and a 20 ms long intense sound stimulus (1 kHz tone burst). In addition, the stimulus was inserted with a silent gap (50 or 20 ms) 100 ms before the onset of the intense sound stimulus.

The sound stimuli were presented as a continuous pure tone background sound [8 kHz with an intensity of 20 dB SL (sensation level)] and an intense sound of 1 kHz with an intensity of 65 dB SL (Fig 1). These frequencies were selected because 1 kHz represents the most sensitive center frequency, and 8 kHz is one of the most frequently reported tinnitus pitches [44]. To account for individual subjects’ hearing levels, the background sound and intense sounds were in the dB SL scale. The better hearing ear at 1 kHz was selected as the stimulus ear. The 1-kHz intense sound stimuli tone bursts were 20-ms long, with five cycles of a rise and a fall each. Effective gap conditions (embedded gap of 50- or 20-ms) for the GPI were selected based on previous acoustic startle response (ASR) and evoked potential studies in humans [12, 14, 17, 23, 36]. From herein, the condition with the preceding gap is referred to as the *gap-intense sound stimulus* and the condition with no preceding gap is referred to as the *no-gap-intense sound stimulus*. In the *gap-intense sound stimulus*, a temporal gap of 50 or 20 ms was inserted into the background sound. The inter-stimulus interval, or time between the end of the temporal gap and the onset of the intense sound, was set to 100 ms. This is considered an optimal interval to elicit sensorimotor- and sensory-gating [23, 45, 46]. To minimize subjects’ predictive behavior, inter-trial intervals between the presentations of the intense sound stimuli were pseudorandomly alternated between one and 3 s. Likewise, sequences of *gap-intense sound stimuli* and *no-gap-intense sound stimuli* were presented pseudorandomly. A total of 100 sound stimuli of each stimulus type were presented. Using the stimulus parameters described above, the duration of the entire measurement process was less than 10 min. To prevent the effect of habituation on the amplitude or inhibition of the ALR, the order of 50- and 20-ms gaps in the retest session was reversed for each subject. For all measurements, a research platform, developed in our laboratory, was used to produce the GPI paradigm stimuli [47]. An ER-2 insert earphone (Etymotic research INC., Elk Grove Village, IL, USA) was used to present stimuli with a flat high-frequency response. A sound level meter (Type 2250 Sound Level Meter, Bruel & Kjaer, Nærum, Denmark) with an ear simulator (IEC Ear Simulator RA0045, G.R.A.S. Sound & Vibration, Holte, Denmark) was used to calibrate all sound outputs at seven frequencies (0.2, 0.5, 1, 2, 4, 6.3, and 8 kHz) in a soundproof booth. The maximum intensity of the intense sound stimulus was 95 dB HL, while the maximum intensity of background sound was 90 dB HL. The maximum producible intensities in the equivalent dB SPL (sound pressure level) were 104- and 103-dB SPL at 1 kHz and 8 kHz, respectively. These were lower than the maximum undistorted outputs (i.e., < 3% third-harmonic distortion) played through the ER-2 insert earphone. The earphone specific outputs were 108- and 112-dB SPL at 1 kHz and 8 kHz, respectively.

### Auditory late response measurement

All measurements were conducted in a soundproof booth. Participants were seated in a reclining armchair and instructed not to move, close their eyes, or fall asleep. They were also told not to pay attention to any sound stimuli (passive listening). Muted clips of historical, scientific, or medicine-based videos were played to divert the subjects’ attention away from the sound stimuli and prevent drowsiness. Highly violent, emotional, or fearful video clips were excluded. Each measurement was followed by a three-minute break; however, for drowsy or fatigued subjects, breaks longer than 3 min were allowed before acquiring the subsequent measurement. To average out the influence of the visual stimuli, visual changes were not synchronized with the sound stimuli. Before attaching electrodes to each participant, we abraded the skin to lower the skin-electrode impedances to below 5 kΩ. Electrodes were placed on the mastoid [ipsilateral (A1 or A2) to the earphone] as a reference electrode, and on the forehead (Fpz) as a ground electrode. Adhesive silver-silver chloride electrodes were used (Kendall™ 100 series, Covidien LLC, Mansfield, MA, USA). One active 10 mm gold cup electrode (F-E5GH, Grass Technologies, Warwick, RI, USA) with conductive paste was placed on the vertex (Cz) as an active electrode [29]. A total of 100 epochs for each *gap-intense sound stimulus* and *no-gap-intense sound stimulus* were recorded in pseudorandom order during each measurement. A single epoch was 700 ms long with the above-mentioned components of sound stimulus and 100-ms pre-stimulus onset time, to allow for baseline corrections. During each of the two sessions (test and retest), two measurements, ~10-minute long each, were taken from each participant–one that contained the 50 ms embedded gaps and the other with the 20 ms embedded gaps. The acquired signals were filtered with an analog band-pass filter with a cutoff frequency of 1 to 100 Hz. The signals then went through analog-digital conversion (ADC) with a sampling frequency of 1 kHz. Further, a digital low-pass Butterworth filter with a cutoff frequency of 30 Hz was used for waveform smoothing. In a single measurement, epochs were ensemble-averaged to produce two auditory late response waveforms corresponding to the *gap-intense sound stimuli* and *no-gap-intense sound stimuli*. Epochs exceeding ± 50 μV, which were observed in less than 10% of all measurements, were excluded.

### Data analysis

Predefined N1 (60–150 ms) and P2 (100–250 ms) acquisition windows were used to create a peak detection algorithm, which computed the amplitudes of the N1-P2 complex. Generally, a negative peak, N1, and a positive peak, P2, were observed at 90–150 ms and 160–200 ms, respectively, after the onset of the intense sound stimulus. Detected peak amplitudes were then visually inspected and confirmed. We then calculated the peak-to-peak amplitude of the N1-P2 complex in response to the *gap-intense sound stimuli* (Gap) and that of the *no-gap-intense sound stimuli* (No-Gap). We also calculated the Gap/No-Gap ratio, defined as the ratio between N1-P2 amplitude during Gap stimulation to that during the No-Gap stimulation. The inhibition deficit was indicated by a high Gap/No-Gap ratio. Offline data processing was performed using MATLAB (R2018b, MathWorks, Natick, MA, USA). First, a two-tailed one-sample *t*-test was performed to determine whether a significant inhibition occurred under each condition. The hypothesized population mean was set to 1.0, meaning there was no difference between Gap and No-Gap responses. Next, we applied a linear mixed-effects model, with the Gap/No-Gap ratio as the dependent variable and the age (continuous variable) and two gap durations (categorical repeated measures) as the independent variables. Pearson correlation coefficients between the age and the Gap/No-Gap ratio for each session were also calculated. For comparison purposes, correlation analysis between age and N1-P2 amplitude in the condition with no preceding gap was performed. The test-retest reliability of the Gap/No-Gap ratio and the N1-P2 amplitude were evaluated by calculating correlation coefficients between sessions. Furthermore, the relationship between age and the hearing thresholds was analyzed because the present study had wide inclusion criteria regarding hearing levels. Pearson correlation coefficients between the hearing threshold and the Gap/No-Gap ratio for each session were also calculated. An independent *t*-test to compare the Gap/No-Gap ratios between male and female subjects was done to analyze the effect of gender, which could be another confounding factor. Last, the trend of the peak-to-peak amplitudes of the responses over the stimulus repetitions for each participant was analyzed to determine whether adaptation occurred in the ALR measurement. Continuous variables are presented as mean ± standard error of the mean (SEM). SPSS (SPSS Statistics v23, IBM SPSS Statistics, Armonk, NY, USA) was used for statistical analysis. Since it was not possible to determine the expected effect size for the linear mixed model, we did not perform a power analysis. However, it should be noted that the sample size required to determine a 0.4 difference in correlation coefficients is approximately 47 (alpha: 0.05, beta:0.2) [48]. Differences were considered significant when *P* < 0.05.

## Results

**Fig 2:**
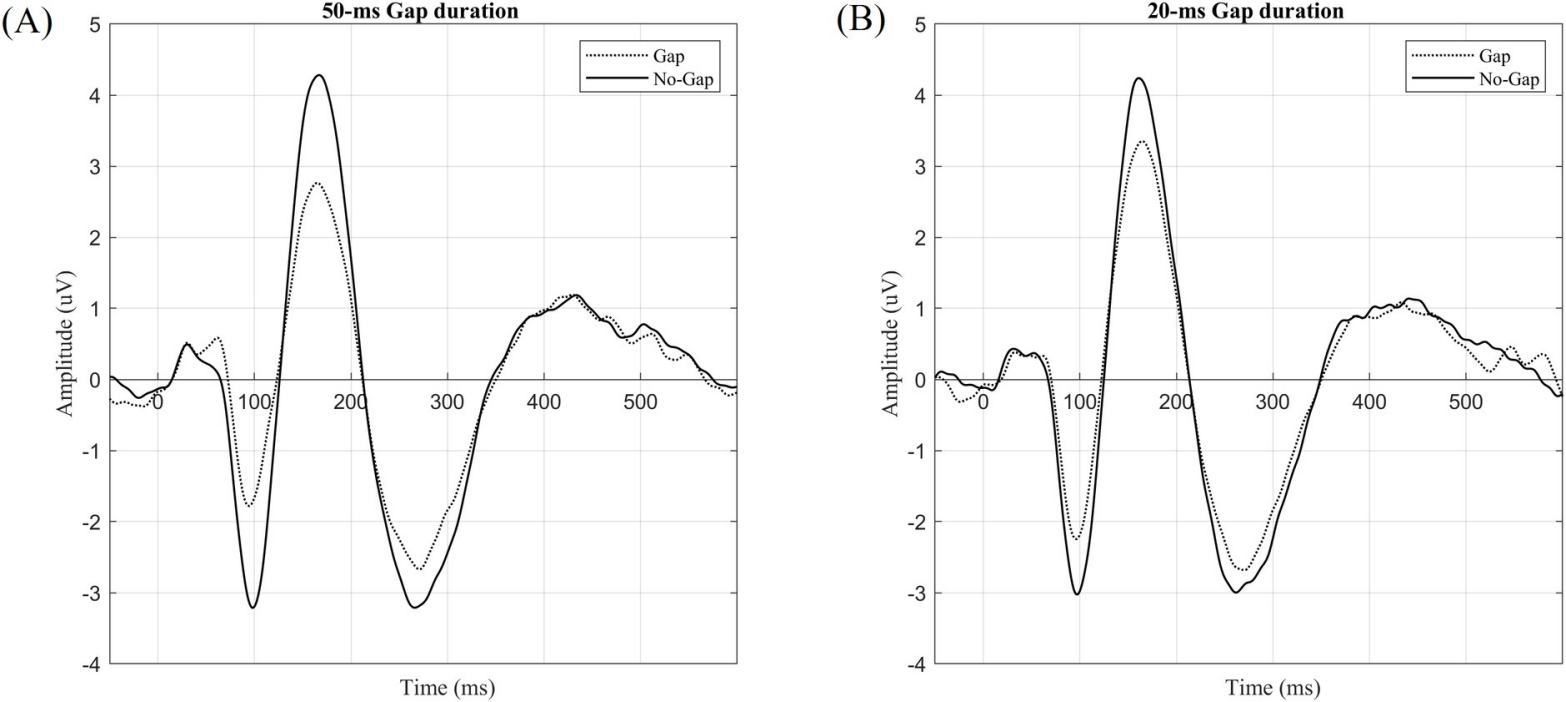
Grand averaging waveforms of the 50 (A) and 20 ms (B) gap durations. Stimulus initiation was set as time zero.

**Fig 3:**
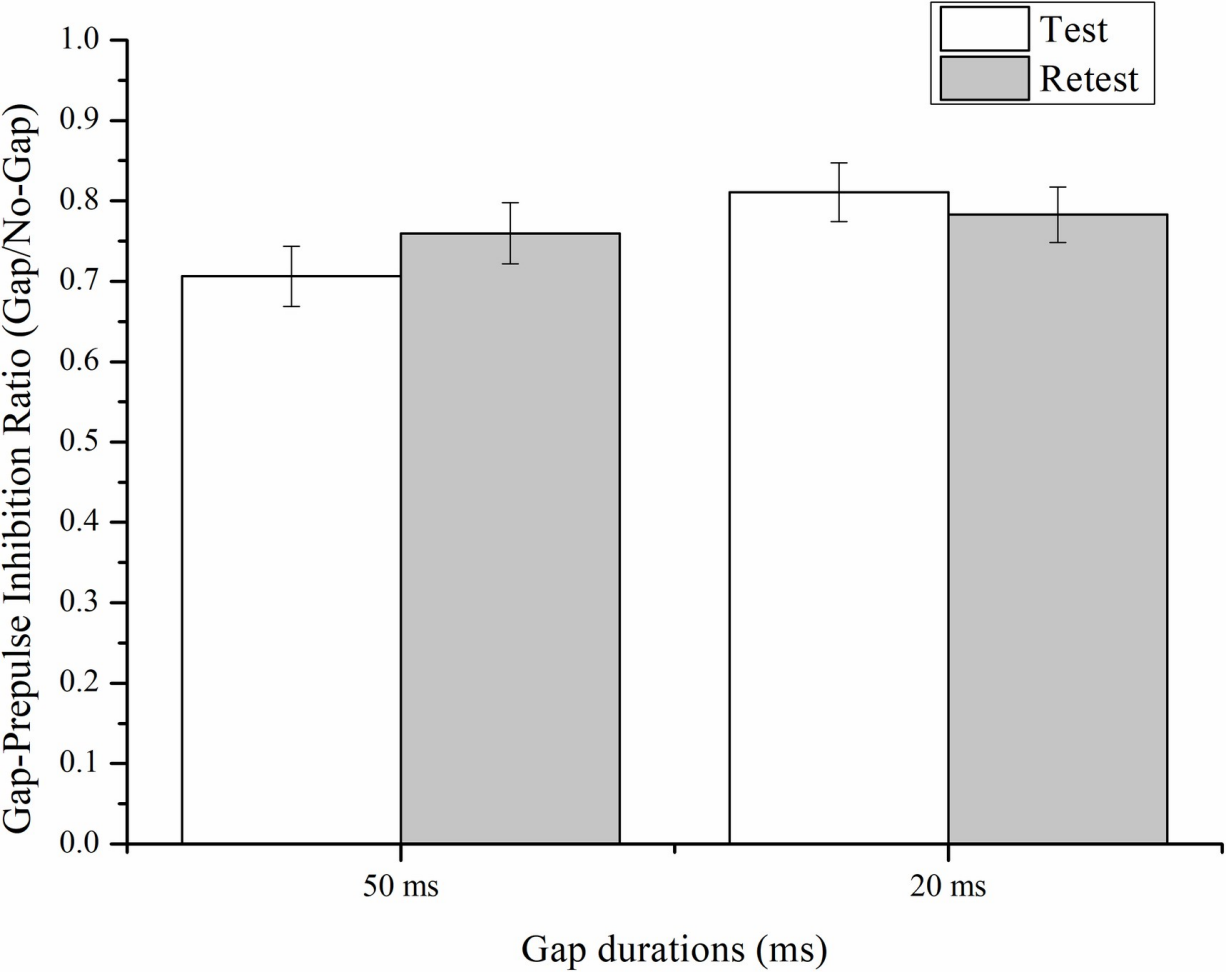
Gap/No-gap ratios with 50-ms and 20-ms gaps in the test and retest sessions. Error bars indicate standard error of the means (SEM).

Fig 2 shows the grand averaged waveforms of the 50 and 20 ms gap durations across all subjects in the test session. The amplitude of auditory cortical responses to the Gap (dotted line) was smaller than that to the No-Gap (solid line). As shown in Fig 3, with a 50-ms gap, the Gap/No-Gap ratios in the test and retest sessions were 0.71 ± 0.04 and 0.76 ± 0.04, respectively. One-sample *t*-tests confirmed significant gap-induced inhibition in both sessions [test: *t*(56) = -7.853, *P* < 0.001; retest: *t*(45) = -6.331, *P* < 0.001]. With a 20-ms gap, the Gap/No-Gap ratios in the test and retest sessions were 0.81 ± 0.04 and 0.78 ± 0.03, respectively. One-sample *t*-tests confirmed significant gap-induced inhibition in both sessions [test: *t*(56) = -5.169, *P* < 0.001; retest: *t*(45) = -6.342, *P* < 0.001].

Although there was a trend for the 20 ms gap to produce less inhibition than the 50 ms gap, the expected main effects of gap length in the two sessions were not significant [test: *F*_1,55_ = 2.159, *P* = 0.147; retest: *F*_1,44_ = 2.29, *P* = 0.137]. However, an interaction between gap length and age was observed in both sessions [test: *F*_2,55_ = 4.663, *P* = 0.013; retest: *F*_2,44_ = 3.831, *P* = 0.029]. That is, Gap/No-Gap ratio significantly increased with age, but only within a certain gap length. On test data, using a range estimate for the standard deviation, the effect of age on the Gap/No-Gap ratio was not significant with a 20-ms gap [*t*(109.7) = 1.38, *P* = 0.17]. However, a significant effect of age on the Gap/No-Gap ratio was found when the gap was set to 50-ms [*t*(109.7) = 2.65, *P* = 0.009]. That is, the Gap/No-Gap ratio significantly increased with age when the gap was 50 ms, but not when it was 20 ms. An identical trend was also found in the retest data. With a 20 ms gap, the effect of age on the Gap/No-Gap ratio was not significant [*t*(86.2) = 0.863, *P* = 0.391], but it was significant with a 50 ms gap [*t*(86.2) = 2.73, *P* = 0.008].

**Fig 4:**
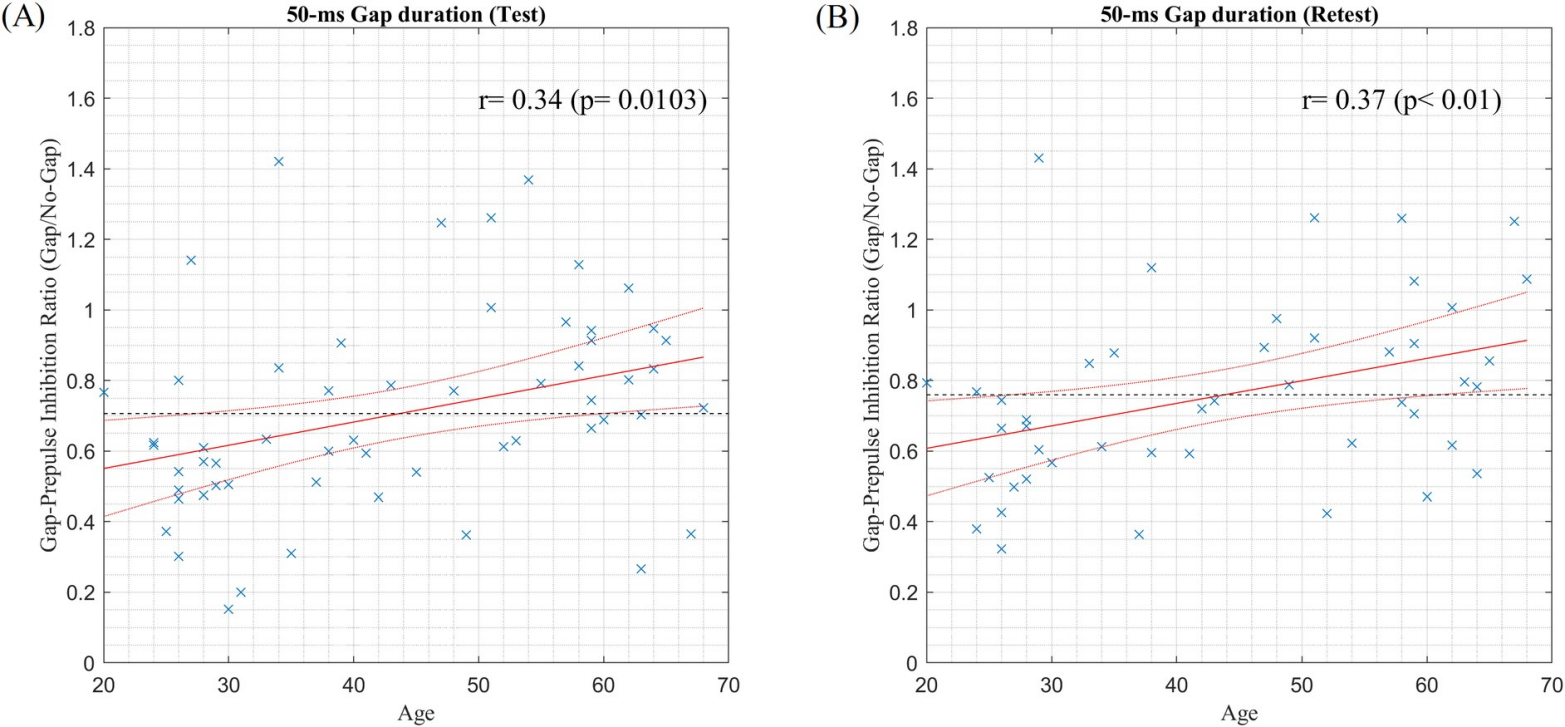
Scatter plots of age *versus* Gap/No-gap ratio with a 50-ms gap duration in the test (A) and retest (B) sessions. The red solid and dotted lines indicate the fitting line and 95% confidence bounds, respectively, based on the linear regression analysis. The black dotted line indicates the average of all Gap/No-gap ratios.

**Fig 5:**
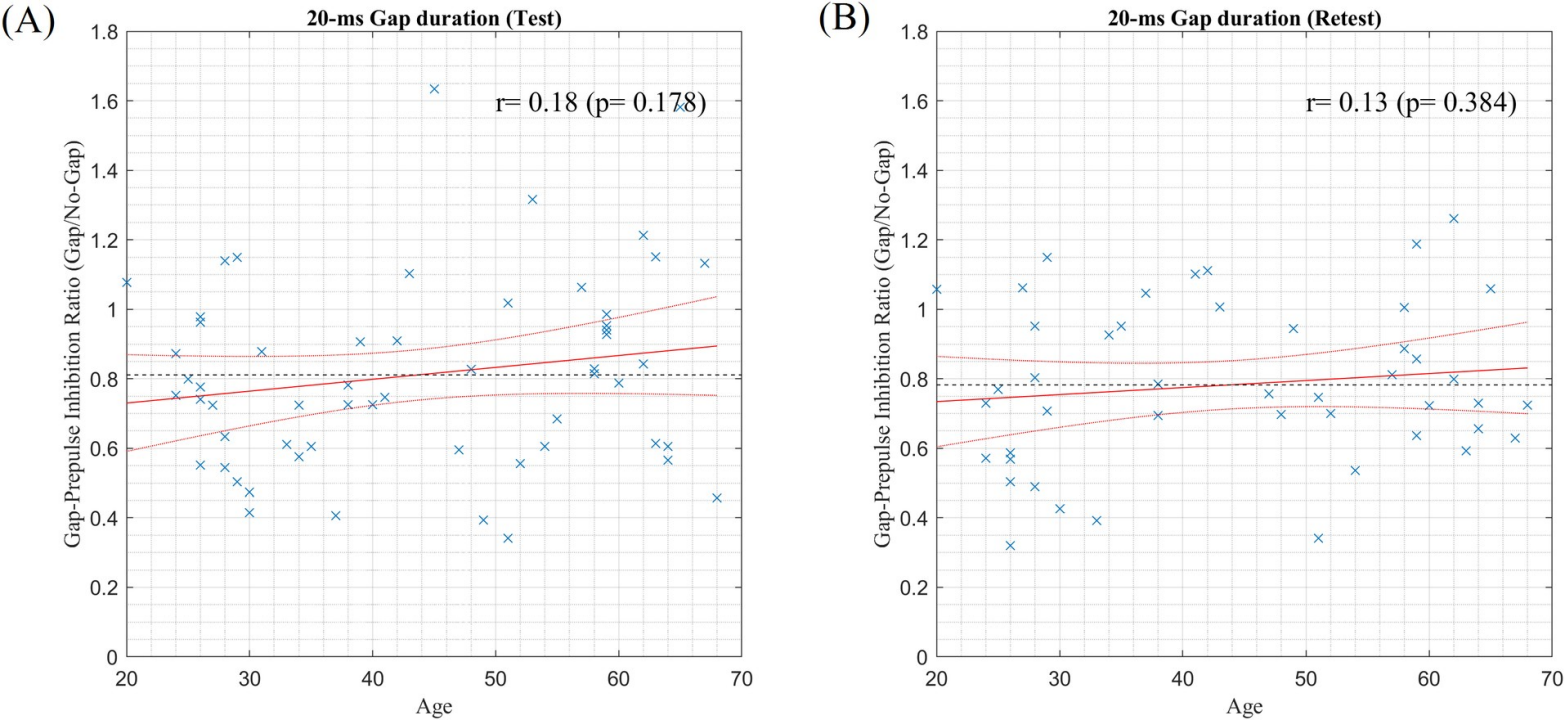
Scatter plots of age *versus* Gap/No-gap ratio with a 20-ms gap duration in the test (A) and retest (B) sessions. The red solid and dotted lines indicate the fitting line and 95% confidence bounds, respectively, based on the linear regression analysis. The black dotted line indicates the average of all Gap/No-gap ratios.

Fig 4 shows scatter plots of the age *versus* the Gap/No-Gap ratio with a 50-ms gap duration. Significant correlations were found in both sessions [test: *r* = 0.34, *P* = 0.0103; retest: *r* = 0.37, *P* < 0.01]. However, no correlations were found with a 20-ms gap duration, as shown in Fig 5 [test: *r* = 0.18, *P* = 0.178; retest: *r* = 0.13, *P* = 0.384].

**Fig 6:**
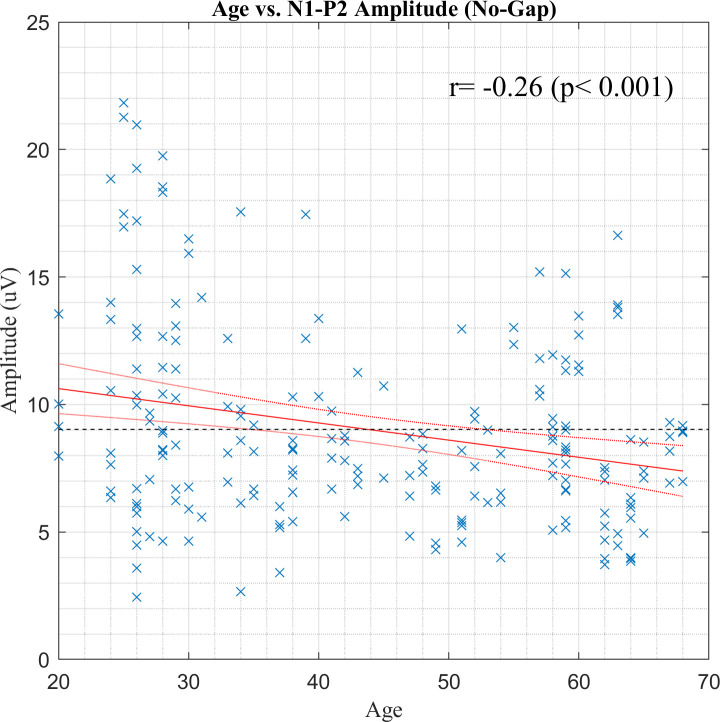
A scatter plot of age *versus* N1-P2 amplitudes in response to *no-gap-intense sound stimuli* in the test and retest sessions. The red solid and dotted lines indicate the fitting line and 95% confidence intervals, respectively, based on linear regression analysis. The black dotted line indicates the average of all N1-P2 amplitudes in response to *no-gap-intense sound stimuli*.

As shown in Fig 6, when plotting all N1-P2 amplitudes in response to *no-gap-intense sound stimulus versus* age, there was a weak but significant trend for younger subjects to have larger amplitudes with a large between-subjects variance [*r* = -0.26, *P* < 0.001].

**Fig 7:**
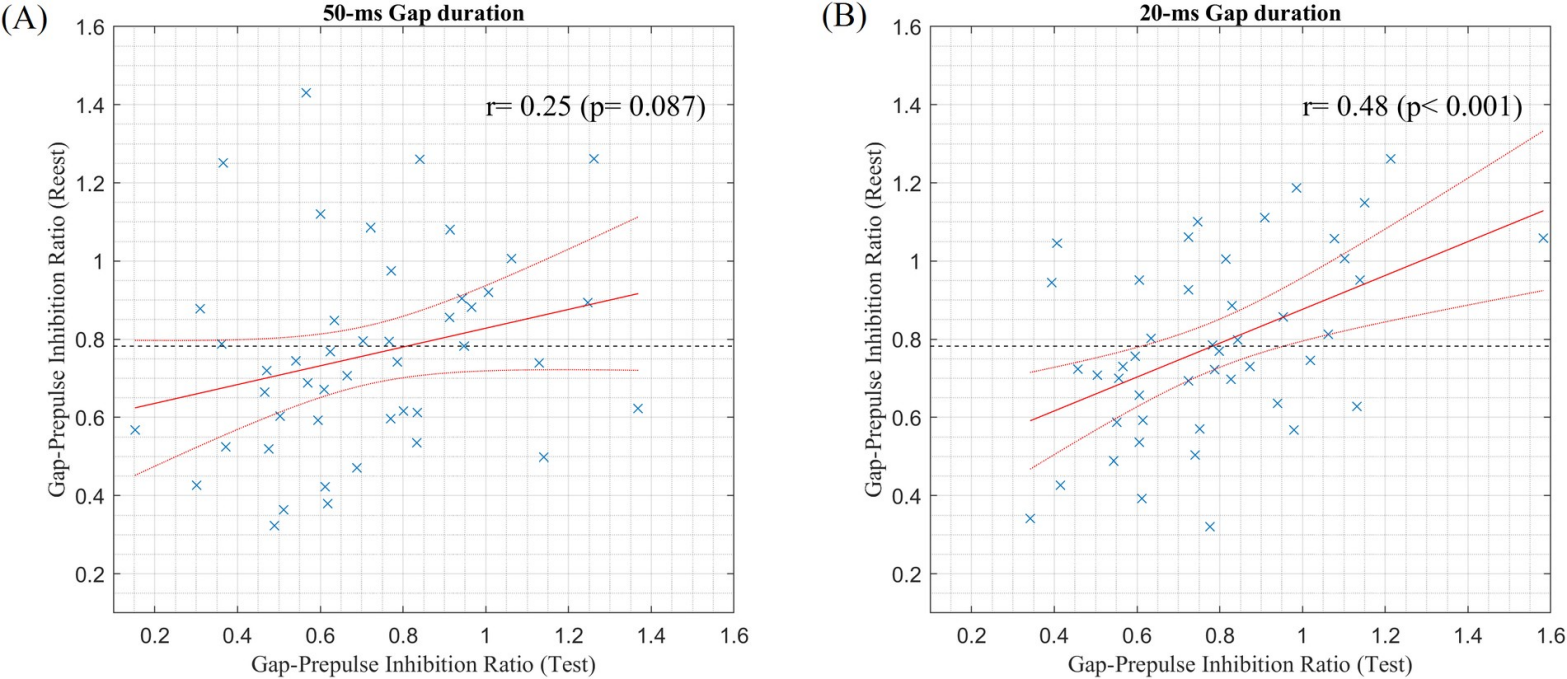
Scatter plots of the Gap/No-Gap ratios between test and retest sessions with 50-(A) and 20-ms (B) gap durations. The red solid and dotted lines indicate the fitting line and 95% confidence intervals, respectively, based on linear regression analysis.

**Fig 8:**
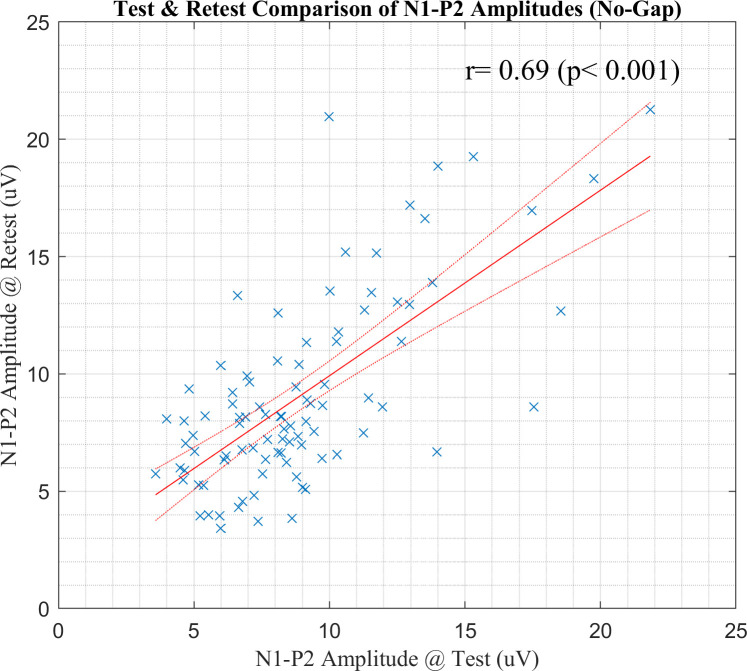
A scatter plot of the N1-P2 amplitudes between test and retest sessions. The red solid and dotted lines indicate the fitting line and 95% confidence intervals, respectively, based on linear regression analysis.

Fig 7 shows scatter plots of the Gap/No-Gap ratios between test and retest sessions with 50- and 20- ms gap durations. The Gap/No-Gap ratios between test and retest sessions had no significant correlation with a 50- ms gap [*r* = 0.25, *P* = 0.087]. With a 20 -ms gap, however, the Gap/No-Gap ratios between test and retest sessions had significant positive correlation [*r* = 0.48, *P* < 0.001]. A significant positive correlation was found in the N1-P2 amplitudes between test and retest sessions as shown in Fig 8 [*r* = 0.69, *P* < 0.001].

**Fig 9:**
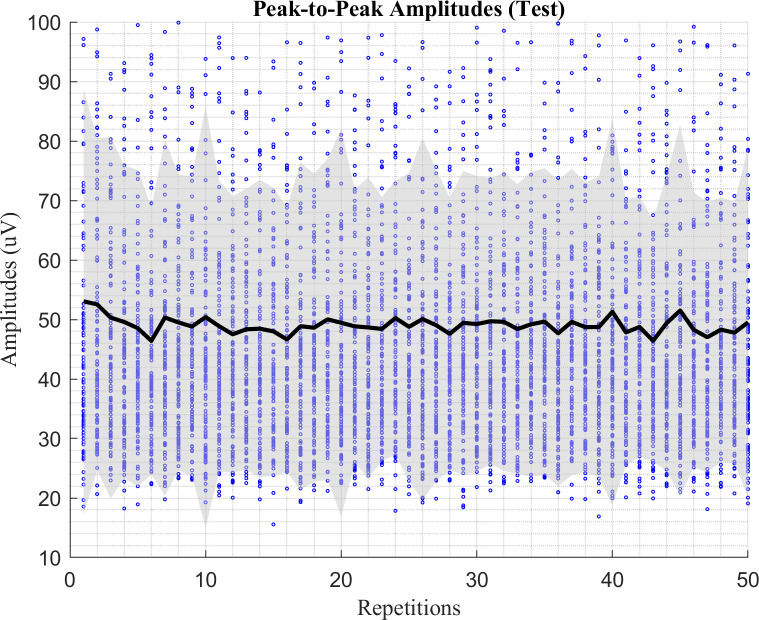
A scatter plot of the peak-to-peak amplitudes of all subjects over the stimulus repetitions in the ALR measurements (test session). The black solid lines indicate the mean of each repetition and the grey-filled area indicates the area under one standard deviation.

The hearing thresholds at 1 and 8 kHz had a significant positive correlations with age [1 kHz: *r* = 0.39, *P* = 0.003; 8 kHz: *r* = 0.59, *P* < 0.001]. No significant correlations between the hearing threshold at 1 kHz and the Gap/No-Gap ratio with a 50 ms gap duration were found in either sessions [test: *r* = 0.04, *P* = 0.751; retest: *r* = 0.01, *P* = 0.935]. No significant correlations were found with a 20 ms gap duration in either session as well [test: *r* = -0.12, *P* = 0.367; retest: *r* = 0.02, *P* = 0.879]. Similarly, no significant correlation between the hearing threshold at 8 kHz and the Gap/No-Gap ratio were found in either sessions [test: *r* = 0.24, *P* = 0.071; retest: *r* = 0.02, *P* = 0.89], as was the case with a 20 ms gap duration in either session [test: *r* = 0.06, *P* = 0.644; retest: *r* = 0.08, *P* = 0.582]. Furthermore, no gender effect was found. With a 50- ms gap, no significant difference were found in either session [test: *t*(55) = -0.452, *P* = 0.653; retest: *t*(44) = -0.342, *P* = 0.734]. With a 20 ms gap, similarly, no significant differences were found in either sessions [test: *t*(55) = -0.893, *P* = 0.376; retest: *t*(44) = -0.414, *P* = 0.681]. In addition, as shown in Fig 9, no systematic decrease of the startle response was observed in the peak-to-peak amplitudes of the responses over stimulus repetitions in any of the subjects in the ALR measurements of the test session.

## Discussion

In the present study, we hypothesized that age could be a crucial confounding factor when investigating the GPI paradigm with a cortical long-latency response as an objective measure of tinnitus. Previous human studies with the GPI paradigm did not look into this factor, focusing on other factors such as the effect of gap durations [12, 17, 36]. We found a significant interaction between age and inhibition of the N1-P2 complex when using the GPI paradigm. The age-related change of the Gap/No-Gap ratio was evident with a longer gap (50 ms) but was absent when a shorter gap (20 ms) was used. These findings were reproducible. Moreover, a significant test-retest reliability of the Gap/No-Gap ratio was found only with a 20 ms gap duration but not with a 50 ms gap duration. The N1-P2 amplitude showed a weak but significant trend in younger subjects to have larger amplitude with the large between-subjects variance even in similar ages. Previous publications have reported discrepancies on age-related amplitude changes of the N1 and P2 waves [49, 50]. However, given the two different gap conditions in each subject, this might not affect the results of the present study. In addition, no effects of hearing loss nor gender on the Gap/No-Gap ratio were found in either the test or retest sessions. From a clinical point of view, this consistent outcome, with one less confounding factor when using the shorter gap (20 ms), could serve as an important basis towards building an objective measure of tinnitus. It is noteworthy that in our previous comparative study that included tinnitus subjects, the 20-ms gap was significantly better than the 50-ms gap in terms of discriminating between subjects affected by tinnitus and the age-matched normal controls. Taken together, the GPI paradigm of the N1-P2 complex, with a 20-ms gap, may present two advantages towards an objective measure of tinnitus: 1) better discriminating ability, 2) lower effect of age, and 3) better reproducibility.

Although we focused on a specific GPI paradigm, our findings related to the 50-ms gap condition are consistent with previous reports on the effect of age on general temporal processing. Temporal processing functions can be analyzed through the conventional gap detection test [51–54]. Prior works have concluded that the detection of short, silent gaps depends on the age of the individual, with older subjects exhibiting higher gap detection thresholds than younger ones [55–60]. This is not surprising when considering the effect of aging on the perceptual and cognitive functions, and the decline in hearing and vision perception that typically begins when people are in their 40s [56, 61]. However, in one previous study on conventional gap detection, the performance of older adults (mean age of 69.8 years) was not different from younger adults (mean age of 24.2 years) when the gap was 12 ms or longer [62]. One possible explanation for this discrepancy is that the GPI paradigm is a passive task, while the conventional gap detection test requires the subject’s attention, which could compensate for pre-attentive processing deficits [63, 64]. Another explanation would be that the GPI paradigm is a more challenging task than the conventional gap detection paradigm because of the intense sound stimuli. The intense sound stimuli that follow the gap make it much more challenging for the subjects to detect the silent gaps. In our previous psychoacoustic study that evaluated the discriminating ability between Gap and No-Gap, the accuracy was only 50%, even with 300-ms-long gaps [65].

Why does age have no effect when using a 20 ms gap? First of all, the 20 ms gap is a more difficult condition in terms of temporal processing. It seems that the 50 ms gap is an easy condition for pre-attentive auditory processing in young subjects who can comfortably detect the gap, resulting in a very strong inhibition. When the subject grows older, the 50 ms gap is not as easily or readily detected. The temporal processing power of older subjects might need to reach its limits to barely detect the gap, resulting in a small inhibition [66, 67]. If the gap is 20 ms long, it seems that the condition is difficult for both young and old. That is, due to a ceiling effect in task difficulty, now the temporal processing power of the young subjects also reaches its limits, resulting in a similar amount of inhibition among all subjects. In a conventional gap detection study, the age effect between younger and older groups was observed only with 6- and 9-ms gaps (easy condition) but not with a 3-ms gap (difficult condition) [62]. The detection rate with the 3-ms gap became the same for both groups, which means that the temporal processing power reached its ceiling in this difficult condition. Another possible explanation is that a shorter gap does not allow for the involvement of age-related higher-level processing. Fourier and Hebert have reported of a significant GPI of the ASR with 25-ms embedded gaps (mean GPI ratio = 63.2%) and even with 5-ms embedded gaps (mean GPI ratio = 75.8%) in relatively young adults (mean ages = 23.7 and 22.1 years, respectively) [12]. Although N1 and P2 components of the ALR are more related to pre-attentive and obligatory sensory events [30], it is known that the PPI could be modulated by higher cortical processes such as attention or cognition (top-down modulation) [68, 69]. In the present study, a shorter gap provided a shorter cue onset timing, which could allow for less time for higher-level processing. This might lead to lower involvement of the age-related temporal processing decline, while the pre-attentive inhibition mechanism is preserved [70].

A further study is needed to assess the influence of age on the GPI in tinnitus patients. The background sound frequency effect should also be investigated because responsiveness to a presented stimulus can be influenced by the associated background frequency [71]. To develop the GPI paradigm toward an objective measure of tinnitus in humans, it would be necessary to investigate appropriate combinations of gap duration and background frequency as a trade-off between sensitivity and specificity.

## Conclusion

We found an interaction between age and the embedded gap duration in the GPI paradigm in association with the pre-attentive cortical-evoked response. The age of the subject had a significant effect on the Gap/No-Gap ratio when the gap was long (50 ms), but not when it was short (20 ms). The ceiling effect in task difficulty is presumed to be the reason for this difference. Furthermore, the shorter onset timing of the inhibition cue might allow for a lower top-down modulation probability. Our findings may contribute to the development of an objective test that could draw a clear distinction between tinnitus patients and healthy controls, ultimately impacting both rigorous research and clinical practice.
